# Real‐world experience of carfilzomib‐associated cardiovascular adverse events: SEER‐Medicare data set analysis

**DOI:** 10.1002/cam4.3568

**Published:** 2020-11-10

**Authors:** Rohit Bishnoi, Zhigang Xie, Chintan Shah, Jiang Bian, Hemant S. Murthy, John R. Wingard, Nosha Farhadfar

**Affiliations:** ^1^ Division of Hematology and Oncology Department of Medicine University of Florida Gainesville FL USA; ^2^ Department of Health Services Research, Management and Policy College of Public Health and Health Professions University of Florida Gainesville FL USA; ^3^ Department of Health Outcomes and Bioinformatics College of Medicine University of Florida Gainesville FL USA; ^4^ Division of Hematology/Oncology Mayo Clinic Alix School of Medicine Mayo Clinic Florida Jacksonville FL USA; ^5^ Bone Marrow Transplant Program University of Florida Health Cancer Center Gainesville FL USA; ^6^ Division of Hematology/Oncology University of Florida College of Medicine Gainesville FL USA

**Keywords:** cardiotoxicity, cardiovascular adverse events, carfilzomib, multiple myeloma, proteasome inhibitors

## Abstract

Carfilzomib was approved for the treatment of multiple myeloma in 2012 and since then there have been concerns for cardiovascular toxicity from its use. With this study, we aim to further study the hazards and underlying risk factors for cardiovascular adverse events associated with carfilzomib. This study was conducted using Surveillance, Epidemiology, and End Results (SEER)‐Medicare data set of multiple myeloma from 2001 to 2015. Data were analyzed for hazards ratio of cardiovascular adverse events between carfilzomib users and nonusers. We identified 7330 patients with multiple myeloma of whom 815 were carfilzomib users. Carfilzomib users had a statistically significant hazard ratio of 1.41 with *p *< 0.0001 for all cardiovascular adverse events as compared to nonusers. Carfilzomib use was significantly associated with increased risk of heart failure (HR 1.47, *p* = 0.0002), ischemic heart disease (HR 1.45, *p* = 0.0002), and hypertension (HR 3.33, *p* < 0.0001), whereas there was no association between carfilzomib use and cardiac conduction disorders (arrhythmia and heart blocks). Carfilzomib users were at higher risk of new‐onset edema (HR 5.09, *p* < 0.0001), syncope (HR 4.27, *p* < 0.0001), dyspnea (HR 1.33, *p* < 0.0001), and chest pain (HR 1.18, *p* < 0.0001) as compared to carfilzomib nonusers. Age above 75 years, preexisting cardiovascular disease, obesity, and twice a week carfilzomib schedule were significant risk factors associated with cardiovascular adverse events in carfilzomib users. The median time of the onset for all cardiovascular adverse events was 3.1 months. This study has identified a significantly higher likelihood of cardiovascular adverse events in elderly Medicare patients receiving carfilzomib.

## Graphical abstract

Carfilzomib associated cardiotoxicity has been reported in trials and is a concern with its use. In this study, from SEER‐medicare datasets that represent real‐world scenarios, we identified that the hazard ratio of 1.41 (*p* < 0.0001) in patients using carfilzomib as compared to non‐users. Carfilzomib use was associated with increased risk of heart failure, ischemic heart disease, and hypertension.

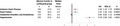

## 1 BACKGROUND

Proteasome inhibitors (PI) comprise an important class of drugs for treating multiple myeloma (MM). Currently, three drugs are approved in this class, bortezomib, carfilzomib, and ixazomib. Carfilzomib is a second‐generation, highly selective and irreversible PI which received accelerated approval by the United States Food and Drug Administration (FDA) in July 2012.^1^ This approval was based on a phase II study where carfilzomib demonstrated an overall response rate (ORR) of about 23% for relapsed and refractory multiple myeloma (RRMM).^2^ Since then carfilzomib has been approved for multiple other indications for the treatment of MM. Presently, as per the National Comprehensive Cancer Network (NCCN) guidelines, carfilzomib is recommended for primary therapy of MM in transplant‐eligible as well as transplant‐ineligible patients. In the RRMM, carfilzomib is recommended as a preferred regimen either as a single agent or in combination with other agents.

Since its approval, cardiovascular adverse events (CVAEs) from carfilzomib has been a concern. Initially, carfilzomib associated CVAEs were reported in about 22% patients from the grouped data of four phase II studies in 2013.^3^ Meta‐analyses of clinical trials have shown that up to 18% of patients experienced CVAEs.^4^, ^5^, ^6^, ^7^ These studies reported CVAEs with variable rates and the most common CVAEs were hypertension (18.5%) and heart failure (6.7%). Arrhythmia (2.4%) and ischemic events (3.7%) were observed less commonly. Other significant CVAEs were dyspnea (31.9%) and edema (24.7%).^6^, ^7^

Multiple studies have tried to identify risk factors for Carfilzomib associated cardiotoxicity. Retrospective studies from single institutions have reported prior cardiac history as a risk factor for carfilzomib associated cardiotoxicity.^8^, ^9^, ^10^ Most of CVAEs have been reported to occur soon after carfilzomib use, rarely beyond 12 cycles and the systolic dysfunction is usually reversible.^9^, ^11^, ^12^ Current evidence is not clear for an association of CVAEs with the dose or the duration of carfilzomib use but re‐challenge with reduced dose has been recommended.^4^, ^11^, ^13^ The prospective observational study by Cornell et al reported that 51% of patients treated with carfilzomib developed CVAEs including heart failure (41%), hypertension (23%), arrhythmia (7%), an acute coronary syndrome in (6%), and also reported chest pain in 9% of patients.^14^ There was no association between drug dose, infusion time, and concurrent drugs or fluids administered with carfilzomib but elevated natriuretic peptide was associated with increased risk of CVAEs. Similarly, 33% of patients experienced CVAEs in the study by Bruno et al and baseline uncontrolled blood pressure, left ventricular hypertrophy, and higher pulse‐wave velocity were identified as risk factors.^15^

Cardiovascular adverse events remains the drug limiting toxicity of carfilzomib and NCCN also alerts for potential carfilzomib related cardiac and pulmonary toxicity, especially in elderly patients. As the median age for diagnosis of MM is 70 years, nearly two‐thirds of patients have preexisting cardiovascular disease at baseline, hence, at risk of developing carfilzomib associated CVAEs.^16^

We have enough scientific evidence about CVAEs from the use of carfilzomib which is mainly obtained through pooled analysis of data from clinical trials. Clinical trials usually exclude patients with preexisting cardiac conditions and the enrolled patients are usually monitored diligently as per clinical trial protocols. Therefore, a clinical trial setting is not the representative of the most commonly encountered scenario in the community clinical practice. This study aims to identify the incidence and risk factors for CVAEs associated with carfilzomib for the treatment of MM using Surveillance Epidemiology and Endpoint Research (SEER)‐Medicare data set, which gives a real‐world experience of carfilzomib.

## 2 METHODS

This study is a retrospective study completed through the SEER‐Medicare data set. The SEER program, supported by the National Cancer Institute (NCI), contains cancer patients’ demographic and tumor characteristics for approximately 34% of the U.S. population. The Medicare data set, maintained by the Centers for Medicare and Medicaid Services, contains health care claims and payment information, for over 97% of the U.S. population aged 65 years or older. We used this linked SEER‐Medicare data set, which captures treatment information after a cancer diagnosis from the Medicare insurance program along with individual patient‐level demographic and survival data from the SEER cancer registry program.

### 2.1 Study population

This study included patients age ≥ 65 years‐old with the diagnosis of MM between 2001 and 2015. To capture the prior history or risk factors for cardiovascular events, only patients who were enrolled in Medicare for at least 1 year before diagnosis were included. Patients were identified using International Classification of Diseases for Oncology, third edition (ICD‐O‐3) codes from the SEER database. We excluded patients with amyloidosis as those patients can have cardiac dysfunction and can be a confounding factor.

ICD 9/10 codes were used to identify past medical history and the new cardiovascular diagnosis after treatment with carfilzomib. Basic demographic data were collected for sex, race/ethnicity, Nicotine/tobacco use, obesity, and Charleston comorbidity index (CCI) among others. Various cardiovascular diagnoses including ischemic heart disease, congestive heart failure, conduction disorders (arrhythmia and blocks) were identified using ICD9/10. We also identified hazards of edema, chest pain, dyspnea, and syncope which are reported separately and not included in CVAEs. Treatment details were identified using Healthcare Common Procedure Coding System (HCPCS) and National Drug Code (NDC) drug codes. Data from Medicare claims for linked patients are available for a year after, up to 2016. Details of the diagnostic codes used for study are available as Table S1.

The primary endpoint of the study was the hazard of the all‐new CVAEs associated with carfilzomib use in the entire study cohort of myeloma patients. The secondary endpoint of the study includes risk factors for CVAEs and hazard of different categories of CVAEs in carfilzomib users. We analyzed the potential risk factors for CVAEs the entire study cohort of myeloma patients and then separately for sub‐group of carfilzomib users. The study focuses mainly on risk factors in carfilzomib users. Impact of carfilzomib use pattern and use of other drugs for treatment of myeloma on were also analyzed. We also calculated the hazard of dyspnea, chest pain, edema, and syncope in carfilzomib users versus nonusers.

### 2.2 Statistical analysis

Patients were divided into two cohorts; carfilzomib users and nonusers. Patient‐, disease‐, and treatment‐related factors were compared using the Chi‐square test for categorical and the Kruskal–Wallis test for continuous variables. A Cox proportional‐hazards model was constructed to determine the relationship between CVAEs and carfilzomib therapy and was controlled for various variables including age, sex, race/ethnicity, previous autologous transplant, CCI, preexisting conditions including body mass index (BMI), nicotine/tobacco use, preexisting diabetes, preexisting hypertension, preexisting cardiovascular conditions, and previous anthracycline use. The goodness of fit was assessed using the method of Hosmer and Lemeshow. Within the treatment group, we also used Cox proportional‐hazards models to examine how the preexisting conditions predicted the newly diagnosed CVAEs. Cox models were also adjusted for the aforementioned patients' characteristics. All statistical tests were two‐sided and statistical significance was defined as *p* < 0.05. Analyses were conducted using SAS version 9.4 software (SAS Institute).

## 3 RESULTS

### 3.1 Patient characteristics

**FIGURE 1:**
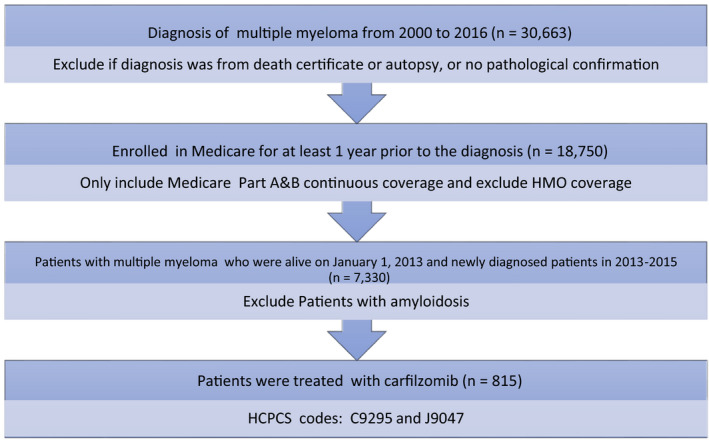
Flowsheet detailing cohort derivation from the SEER‐Medicare data set. HCPCS, Healthcare Common Procedure Coding System; HMO, Health Maintenance Organization

A total of 7330 patients with multiple myeloma were included in the study; 815 (11.1%) carfilzomib users and 6515 (88.9%) carfilzomib nonusers. Figure 1 shows the flowsheet for the study cohort derivation.

Baseline characteristics of the entire cohort including carfilzomib users and nonusers are shown in Table 1. Carfilzomib‐user cohort was younger, white race, had lower CCI compare to nonusers. A higher proportion of carfilzomib users had a history of hypertension or pre‐existing cardiovascular diseases. Furthermore, Carfilzomib use was higher in patients with relapsed myeloma and prior history of stem cell transplant (Table S2). No significant differences were observed between the two groups in terms of sex and pre‐existing history of diabetes. The study model was controlled for these different variables.

**TABLE 1:** Patient demographics and clinical characteristics of carfilzomib users and non‐users

Characteristics	Total	Carfilzomib use				*p*‐value
		Yes	%	No	%	
Year of diagnosis						
2001–2012	4459	537	12.0	3922	88.0	0.0001
2013	1088	121	11.1	967	88.9	
2014	856	92	10.7	764	89.3	
2015	927	65	7.0	862	93.0	
Age groups						
66–69	1813	286	15.8	1527	84.2	<0.0001
70–74	2015	291	14.4	1724	85.6	
75–79	1627	146	9.0	1481	91.0	
80+	1875	92	4.9	1783	95.1	
Sex						
Male	3846	429	11.2	3417	88.8	0.9185
Female	3484	386	11.1	3098	88.9	
Race/ethnicity						
Non‐Hispanic white	5332	640	12.0	4692	88.0	<0.0001
Non‐Hispanic black	1076	98	9.1	978	90.9	
Other	400	29	7.3	371	92.8	
Hispanic	522	48	9.2	474	90.8	
Myeloma						
New	5621	355	6.3	5266	93.7	<0.0001
Relapsed	1709	460	26.9	1249	73.1	
Previous transplant						
No	6483	606	9.3	5877	90.7	<0.0001
Yes	847	209	24.7	638	75.3	
Charleston comorbidity index						
0	4788	613	12.8	4175	87.2	<0.0001
1	1293	129	10.0	1164	90.0	
2	631	41	6.5	590	93.5	
3+	618	32	5.2	586	94.8	
Body mass index						
Other	6775	693	10.2	6082	89.8	<0.0001
Overweight	60	15	25.0	45	75.0	
Obesity	495	107	21.6	388	78.4	
Nicotine/tobacco use						
Never	6565	557	8.5	6008	91.5	<0.0001
Current/former	765	258	33.7	507	66.3	
Pre‐existing diabetes						
No	4311	465	10.8	3846	89.2	0.2795
Yes	3019	350	11.6	2669	88.4	
Pre‐existing hypertension						
No	6682	584	8.7	6098	91.3	<0.0001
Yes	648	231	35.6	417	64.4	
Pre‐existing cardiovascular conditions						
No	3452	302	8.7	3150	91.3	<0.0001
Yes	3878	513	13.2	3365	86.8	
Previous anthracycline use						
No	7177	755	10.5	6422	89.5	<0.0001
Yes	153	60	39.2	93	60.8	

### 3.2 Risk factors associated with CVAEs in the entire study cohort of myeloma patients, including carfilzomib users and nonusers

Based on the multivariate analysis, carfilzomib use was independently associated with an increase in the risk of development of CVAEs in the entire study cohort of MM patients. Compared to carfilzomib nonusers, the HR for CVAEs for carfilzomib users was 1.41 (95% CI 1.26–1.58, *p* < 0.0001). In addition to exposure to carfilzomib, advancing age, male sex, white race, higher CCI, higher BMI, nicotine/tobacco use, preexisting hypertension, and other cardiovascular diagnosis were associated with an increased risk for CVAEs. Pre‐existing diabetes and previous anthracycline use were not associated with higher CVAEs. Please see Table S3 for results of multivariable analysis of the entire study cohort.

### 3.3 Risk factors associated with CVAEs in sub‐group of carfilzomib users

**FIGURE 2:**
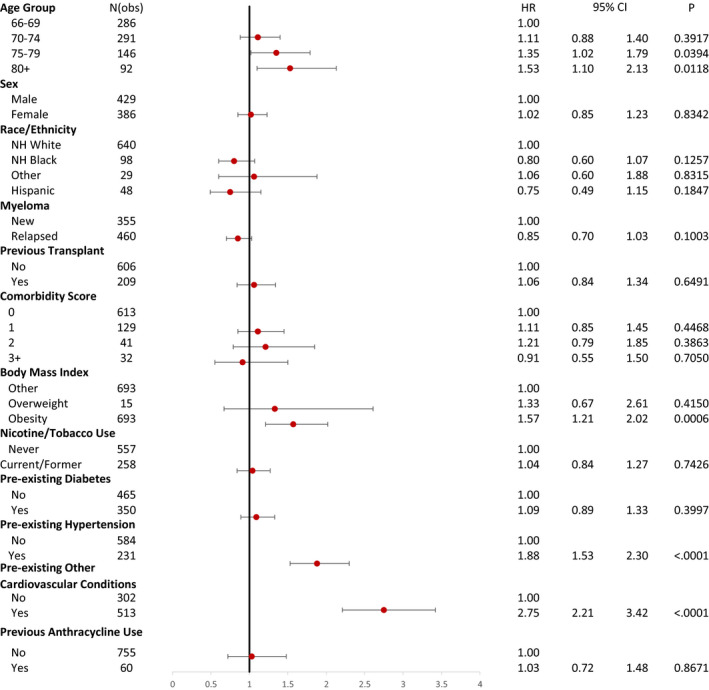
Adjusted hazard ratios for cardiovascular adverse events in carfilzomib users (*n* = 815). CI, confidence interval; HR, hazards ratio; NH, non‐Hispanic

Based on the multivariate analysis, age 75–79 years (HR 1.35, *p* = 0.0394) and above 80 (HR 1.53, *p* = 0.0118) were associated with a significantly higher risk of CVAEs compared to patients aged 65–69 years. Whereas obesity (HR 1.57, *p* = 0.0006), pre‐existing hypertension (HR 1.57, *p* = 0.0006), and preexisting other cardiovascular diagnoses (HR 2.75, *p* < 0.0001) were also significant risk factors. Patient's sex, race/ethnicity, nicotine/tobacco use, preexisting diabetes, previous anthracycline use, and history of the previous autologous transplant were not associated with higher CVAEs. Please see Figure 2 for details. Among all carfilzomib users, 57.6% (*n* = 469) were noted to have one or more CVAEs. The median time to onset of these CVAEs was 3.1 months.

### 3.4 Categories of CVAEs

**FIGURE 3:**
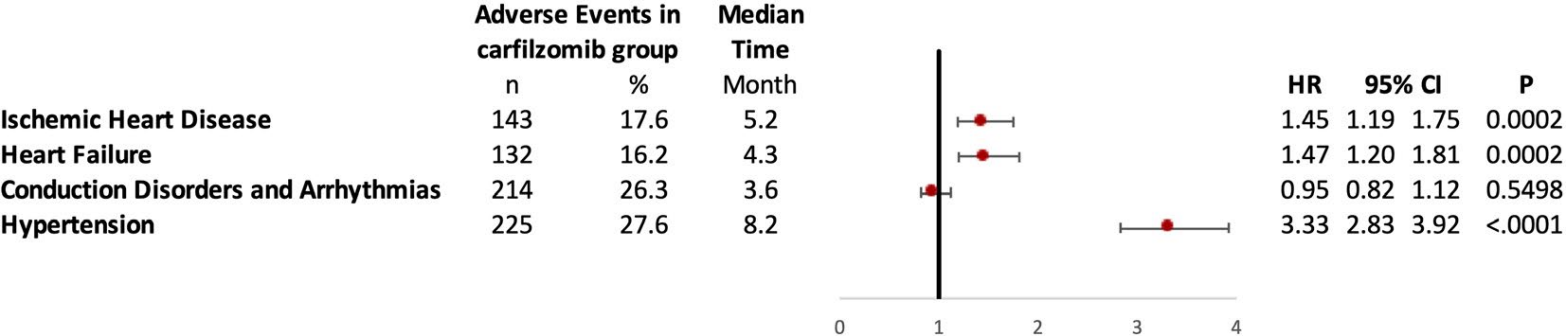
Adjusted hazard ratios of patient using carfilzomib in cardiovascular adverse effects. CI, confidence interval; HR, hazards ratio

The study cohort was then analyzed for the hazards of various categories of new‐onset CVAEs. Cardiovascular events were categorized into ischemic heart disease, heart failure, conduction disorders (arrhythmia and heart blocks), and hypertension. Carfilzomib use was significantly associated with increased risk of heart failure (HR 1.47, *p* = 0.0002), ischemic heart disease (HR 1.45, *p* = 0.0002), and hypertension (HR 3.33, *p* < 0.0001), whereas there was no association between carfilzomib use and cardiac conduction disorders (arrhythmia and heart blocks). Please see Figure 3 for details.

### 3.5 Symptoms

**FIGURE 4:**
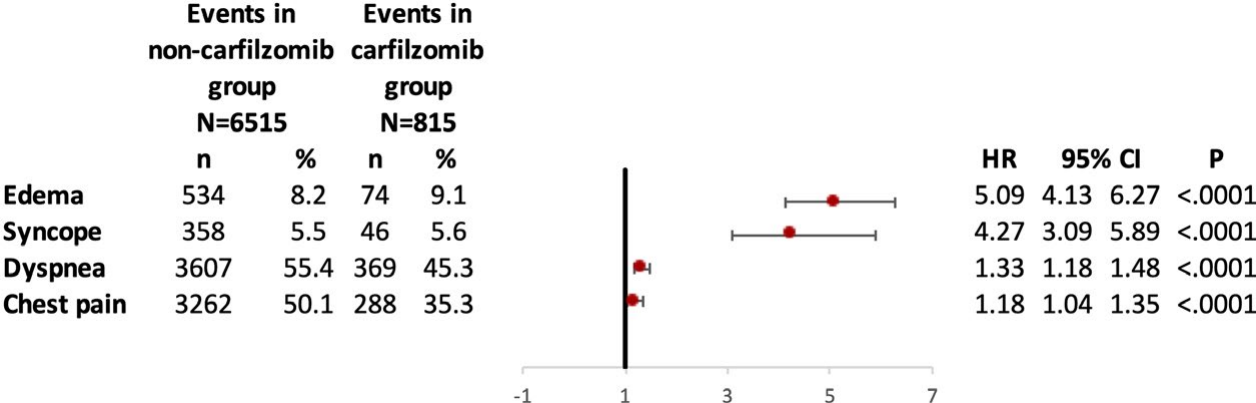
Hazards of new‐onset symptoms in carfilzomib users as compared to nonusers. CI, confidence interval; HR, hazards ratio

Hazards of certain new‐onset symptoms were also studied in the entire study cohort. Carfilzomib users were at higher risk of new‐onset edema (HR 5.09, *p* < 0.0001), syncope (HR 4.27, *p* < 0.0001), dyspnea (HR 1.33, *p* < 0.0001), and chest pain (HR 1.18, *p* < 0.0001) as compared to carfilzomib nonusers (Figure 4).

### 3.6 Carfilzomib use pattern

The SEER‐Medicare data set has limited information about drug doses. We analyzed carfilzomib usage information based on reimbursement patterns. Although, not perfect but this does provide some insight into the pattern of carfilzomib use and associated toxicity. The multivariate logistic regression model was used after controlling various variables as described previously.

The overall median duration of carfilzomib therapy was 3.6 months and the median number of carfilzomib doses used was 24. The median interval between the two doses was 4.5 days. Patients receiving twice a week carfilzomib were identified as a treatment interval of fewer than 4 days. Carfilzomib was used once a week in 38% of patients while in rest 62% of patients; carfilzomib was used twice a week. Weekly infusion of carfilzomib was associated with a lower risk of cardiovascular toxicity compare to twice‐weekly infusions (HR 0.76, 95% CI: 0.62–0.92, *p* = 0.0051).

Carfilzomib was used as a single agent in 61% (*n* = 496) and in combination with other drugs in 39% (*n* = 319) of patients. When prescribed in combination, carfilzomib was most commonly used with cyclophosphamide in 27.7% (*n* = 218) followed by daratumumab in 8.3% (*n* = 68) and Lenalidomide in 2.7% (*n* = 22). Combination use with Pomalidomide or Thalidomide was minimal. In comparison to single‐agent carfilzomib, combined use with cyclophosphamide, daratumumab, lenalidomide, pomalidomide, or thalidomide was not associated with increased risk for CVAEs.

## 4 DISCUSSION

Our study is an extensive review of carfilzomib associated CVAEs using the SEER‐Medicare data set that approximately includes 34% of the U.S. population and represents a commonly encountered patient population as compared to selective patients enrolled through clinical trials. We identified a significantly increased risk of CVAEs with carfilzomib therapy, even after controlling for multiple variables. The incidence of CVAEs (57.6%) was higher as compared to previously reported incidence through pooled analysis but these rates are closer to the prospective study done by Cornell et al to identify the incidence of CVAEs.^14^ Similar to previous retrospective studies, we found that pre‐existing hypertension and other cardiovascular diagnoses were associated with a higher risk of CVAEs after carfilzomib use. Elderly patients above age 75 years and obesity were at higher risk for CVAEs from carfilzomib use. The majority of MM patients who received carfilzomib had relapsed disease and were likely to have received previous treatments or underwent an autologous transplant. Our study did not find that patients with either relapsed disease or previous autologous transplant had higher CVAEs after carfilzomib use.

As previously reported in the literature, we also identified highly significant HR for heart failure, ischemic heart disease, and hypertension while there was no association with conduction disorders (arrhythmia and blocks). Conduction disorders have been reported with carfilzomib use, but the literature review was not consistent with this toxicity. Like other studies^6^, ^12^ our study also identified that carfilzomib users had significant hazards of new‐onset dyspnea, chest pain, edema, and syncope, as compared to nonusers. Although these symptoms are not specific, we recommend careful monitoring of these symptoms as it might help in the early detection of CVAEs.

Our study did not identify any combination regimens to be associated with a higher risk for CVAEs as compared to the use of carfilzomib alone. Once a week use was associated with lower CVAEs as compared to twice a week use of carfilzomib. A study by Moreau et al reported prolonged progression‐free survival and fewer cardiac failure events with the once weekly schedule as compared to a twice‐weekly schedule.^17^ Since studies so far have not shown any clear evidence of dose‐dependent cardiovascular toxicity form carfilzomib, this finding suggests that quicker frequency may be related to higher toxicity. Although this finding is significant, we advise caution in clinical interpretation in the absence of exact dosages information, which unfortunately is a known limitation of the SEER‐Medicare data set.

Various preventative and management strategies have been proposed for carfilzomib‐induced cardiotoxicity by single‐center studies.^18^, ^19^ These include baseline cardiac function evaluation, optimizing underlying hypertension and cardiac conditions, careful fluid management, and symptom directed workup while on carfilzomib therapy. Although faster infusion rates of carfilzomib have been reported to be associated with higher CVAEs the prospective observational study by Cornell et al did not find any such association.^14^, ^20^ Cautious re‐challenge with a reduced dose of carfilzomib after the resolution of cardiac events has also been recommended. European Myeloma network and the Italian society of arterial hypertension also released a consensus paper with a scoring system based on risk factors and management recommendations for patients using carfilzomib.^21^

In summary, with our study results and previous data, we can assertively say that there is enough evidence to strongly associate the significant risk of CVAEs with the use of carfilzomib. As such, there is a need for identifying patients at higher risk, cautious monitoring, prompt identification, and management of carfilzomib associated CVAEs. Patients with pre‐existing cardiovascular conditions should be monitored closely by a multidisciplinary team from cardiology and oncology which is also a recommendation from the International Cardio‐Oncology Society (ICOS).^22^ This is absolutely necessary until we have a full understanding of all the risk factors, preventative measures, and long‐term toxicity from carfilzomib use.

## 5 CONCLUSIONS

This study from a large SEER‐Medicare data set provides further evidence of carfilzomib‐associated CVAEs and identifies potential risk factors.

### 5.1 Study limitations

This study has an inherent limitation of retrospective design and thus we infer association and not direct causation. Although the SEER database includes data from 19 different geographical areas covering approximately 34% of the U.S. population from diverse demographics and locations it cannot be ascertained that every population group has proper representation. Authors caution that results might be affected by various local risk factors, including access to health care, and should be considered for an individual patient The SEER‐Medicare database does not contain clinical measures of disease severity, information regarding chemotherapy dosage and schedule or management of cardiovascular toxicity. In addition, this study included elderly patients with age ≥ 65 years and prone to reporting bias. Therefore, the results may not be generalizable to younger populations or those not covered by Medicare. We have included nicotine/tobacco use and BMI in analyses based on ICD‐9/10 diagnoses codes from Medicare data. However, the sensitivity of these codes is low. Even considering these limitations, studies from SEER‐Medicare data sets have provided clinically relevant information, which often is not feasible from clinical trials.

## CONFLICT OF INTEREST

The authors declare that they have no conflict of interest.

## ETHICS APPROVAL AND CONSENT TO PARTICIPATE

The Institution Review Board (IRB) at the University of Florida, Gainesville FL, approved this study and all standard ethical guidelines were followed. A full waiver of informed consent was obtained.

## CONSENT TO PUBLICATION

Not applicable. The manuscript does not contain any individual patients' data.

## Supporting information

Table S1‐S3

## Data Availability

The data sets used for the current study are available from SEER‐Medicare. This study used the linked SEER‐Medicare database. The interpretation and reporting of these data are the sole responsibility of the authors. The authors acknowledge the efforts of the National Cancer Institute; the Office of Research, Development and Information, CMS; Information Management Services (IMS), Inc.; and the Surveillance, Epidemiology, and End Results (SEER) Program tumor registries in the creation of the SEER‐Medicare database.
