# Supplementary material for: Real‐world experience of carfilzomib‐associated cardiovascular adverse events: SEER‐Medicare data set analysis
Source: Cancer Med. 2020 Nov 10;10(1):70–8. doi: 10.1002/cam4.3568 (PMC7826471; doi:10.1002/cam4.3568)
Supplement: Supplementary file 1 — Table S1‐S3 [file CAM4-10-70-s001.docx]

**Supplementary tables:**

| Hypertension | 401.10, 401.90, 405.1, 405.9, I10, I15.0, I15.1, I15.2, I15.8, I15.9, 401.00, 405.00, I16.0, I16.1, I16.9, 796.2, R03.0 |
| --- | --- |
| Obesity/overweight | E66.1, E66.2, E66.3, E66.8, E66.9, Z68.41, Z68.42, Z68.43, Z68.44, Z68.45, 278.00, 278.01, 278.02, 278.03, V85.41, V85.42, V85.43, V85.44, V85.45 |
| Nicotine/tobacco use | F17.2, Z72.0, Z87.891, 305.1, V15.82 |
| Ischemic heart disease | 413.00, 413.10, 413.90, I20.0, I20.1, I20.8, I20.9, 410.00, 410.20, 410.30, 410.40, 410.10, 410.50, 410.60, 410.70, 410.80, 410.90, 411.00, 411.10, 411.81, 411.89, I21.01, I21.02, I21.09, I21.11, I21.19, I21.21, I21.29, I21.3, I21.4, I21.9, I21.A1, I21.A9, I22.0, I22.1, I22.2, I22.8, I22.9, I23.0, I23.1, I23.2, I23.3, I23.4, I23.5, I23.6, I23.7, I23.8, I24.0, I24.1, I24.8, I24.9, 414.00, 414.10, 414.20, 414.30, 414.40, 414.80, 414.90, I25.10, I25.110, I25.111, I25.118, I25.119, I25.2, I25.3, I25.41, I25.42, I25.5, I25.6, I25.7, I25.8, I25.9 |
| Conduction disorders (arrhythmia and blocks) | 427.31, 427.31, 427.32, I48.0, I48.1, I48.2, I48.3, I48.4  I48.9, 427.41, 427.41, 427.42, 427.50, I49.0, I46.2, I46.8, I46.9, 427.81, 427.00, 427.10, 427.20, 427.60, 427.90, I47.0, I47.1, I47.2, I47.9, I49.1, I49.2 I49.3, I49.4, I49.5, I49.8, I49.9, 426.00, 426.10, 426.20, 426.30, 426.40, 426.50, 426.60, 426.70, 426.81, 426.90, I44.0, I44.1, I44.2, I44.3, I44.4, I44.5, I44.60, I44.69, I44.7, I45.0, I45.1, I45.2, I45.3, I45.4, I45.5, I45.6, I45.8, I45.9 |
| Heart failure | 428.20, 428.20, 428.21, 428.22, 428.23, I50.2, I50.20, I50.21, I50.22, I50.23, 428.30, 428.30, 428.31, 428.32, 428.33, I50.3,  I50.30, I50.31, I50.32, I50.33, 428.40, 428.40, 428.41, 428.42, 428.43, I50.4, I50.40, I50.41, I50.42, I50.43, 428.00, 428.90, 428.10, I50.8, I50.810, I50.811, I50.812, I50.813, I50.814, I50.82, I50.83, I50.84, I50.89, I50.1, I50.9 |
| Cardiomyopathy and other cardiac diagnosis | 425.00, 425.10, 425.20, 425.30, 425.40, 425.50, 425.70, 425.80, 425.90, I42.0, I42.1, I42.2, I42.3, I42.4, I42.5, I42.6, I42.7, I42.8, I42.9, I43, 402.00, 402.01, 402.10, 402.11, 402.90, 402.91,  404.00, 404.01, 404.02, 404.03, 404.10, 404.11, 404.12, 404.13, 404.90, 404.91, 404.92, 404.93, I11.0, I11.9, I13.0, I13.10, I13.11, 429.70, 429.00, 429.10, 429.20, 429.30, 429.40, 429.50, 429.60,  429.81, 429.82, 429.83, 429.89, 429.90, 420.00, 420.90, 422.00  422.90, 423.00, 423.10, 423.20, 423.30, 423.80, 423.90, I30.0, I30.1, I30.8, I30.9, I40.0, I40.1, I40.8, I40.9, I41, I51.0, I51.1, I51.2, I51.3, I51.4, I51.5, I51.7, I51.8, I51.9, I52 |
| Symptom codes  Dyspnea, chest pain, syncope, edema | 780.20, R55, R07.1, R07.2, R07.81, R07.82, R07.89, R07.9, 786.50, 786.51, 786.52, 786.59, 782.30, R60.0, R60.1, R60.9, 786.00, 786.01, 786.02, 786.03, 786.04, 786.05, 786.06, 786.07, 786.09, R06.00, R06.01, R06.02, R06.03, R06.09 |

Table s1: List of ICD 9/10 codes used for cohort derivation and analysis.

| Characteristics | OR | 95% CI | | P-value |
| --- | --- | --- | --- | --- |
| Year of diagnosis |  |  |  |  |
| 2001-2012 | 1.00 |  |  |  |
| 2013 | 1.04 | 0.80 | 1.35 | 0.7647 |
| 2014 | 0.95 | 0.70 | 1.28 | 0.7226 |
| 2015 | 0.47 | 0.33 | 0.66 | <.0001 |
| Age groups |  |  |  |  |
| 66-69 | 1.00 |  |  |  |
| 70-74 | 1.00 | 0.81 | 1.24 | 0.9973 |
| 75-79 | 0.78 | 0.60 | 1.01 | 0.0543 |
| 80+ | 0.45 | 0.34 | 0.60 | <.0001 |
| Sex |  |  |  |  |
| Male | 1.00 |  |  |  |
| Female | 1.17 | 0.98 | 1.39 | 0.0826 |
| Race/ethnicity |  |  |  |  |
| Non-Hispanic White | 1.00 |  |  |  |
| Non-Hispanic Black | 0.70 | 0.54 | 0.91 | 0.0084 |
| Other | 0.75 | 0.49 | 1.16 | 0.1935 |
| Hispanic | 0.87 | 0.61 | 1.25 | 0.4566 |
| Myeloma |  |  |  |  |
| New | 1.00 |  |  |  |
| Relapsed | 5.14 | 4.28 | 6.17 | <.0001 |
| Previous transplant |  |  |  |  |
| No | 1.00 |  |  |  |
| Yes | 1.71 | 1.36 | 2.16 | <.0001 |
| Charleston comorbidity index |  |  |  |  |
| 0 | 1.00 |  |  |  |
| 1 | 0.63 | 0.49 | 0.81 | 0.0003 |
| 2 | 0.40 | 0.27 | 0.60 | <.0001 |
| 3+ | 0.22 | 0.14 | 0.35 | <.0001 |
| Body mass index |  |  |  |  |
| Other | 1.00 |  |  |  |
| Overweight | 3.21 | 1.52 | 6.80 | 0.0023 |
| Obesity | 2.12 | 1.57 | 2.87 | <.0001 |
| Nicotine/tobacco use |  |  |  |  |
| Never | 1.00 |  |  |  |
| Current/former | 6.84 | 5.41 | 8.63 | <.0001 |
| Pre-existing diabetes |  |  |  |  |
| No | 1.00 |  |  |  |
| Yes | 1.27 | 1.05 | 1.53 | 0.0157 |
| Pre-existing hypertension |  |  |  |  |
| No | 1.00 |  |  |  |
| Yes | 7.02 | 5.60 | 8.80 | <.0001 |
| Pre-existing cardiovascular conditions |  |  |  |  |
| No | 1.00 |  |  |  |
| Yes | 1.64 | 1.36 | 1.96 | <.0001 |
| Previous anthracycline use |  |  |  |  |
| No | 1.00 |  |  |  |
| Yes | 3.55 | 2.37 | 5.33 | <.0001 |

Table s2: Multivariable logistic regression for the study cohort to identify the factors associated with carfilzomib use.

| Characteristics | HR | 95% CI | | P-value |
| --- | --- | --- | --- | --- |
| Carfilzomib |  |  |  |  |
| No | 1.00 |  |  |  |
| Yes | 1.41 | 1.26 | 1.58 | <.0001 |
| Age groups |  |  |  |  |
| 66-69 | 1.00 |  |  |  |
| 70-74 | 1.18 | 1.08 | 1.28 | 0.0001 |
| 75-79 | 1.24 | 1.13 | 1.35 | <.0001 |
| 80+ | 1.55 | 1.41 | 1.69 | <.0001 |
| Sex |  |  |  |  |
| Male |  |  |  |  |
| Female | 0.87 | 0.82 | 0.93 | <.0001 |
| Race |  |  |  |  |
| Non-Hispanic White | 1.00 |  |  |  |
| Non-Hispanic Black | 0.86 | 0.79 | 0.94 | 0.0005 |
| Other | 0.92 | 0.81 | 1.05 | 0.2035 |
| Hispanic | 0.85 | 0.75 | 0.95 | 0.0059 |
| Myeloma |  |  |  |  |
| New | 1.00 |  |  |  |
| Relapsed | 1.03 | 0.95 | 1.11 | 0.4942 |
| Previous transplant |  |  |  |  |
| No | 1.00 |  |  |  |
| Yes | 0.98 | 0.89 | 1.08 | 0.6828 |
| Charleston comorbidity index |  |  |  |  |
| 0 | 1.00 |  |  |  |
| 1 | 1.31 | 1.20 | 1.42 | <.0001 |
| 2 | 1.59 | 1.43 | 1.76 | <.0001 |
| 3+ | 1.82 | 1.63 | 2.03 | <.0001 |
| Body mass index |  |  |  |  |
| Other | 1.00 |  |  |  |
| Overweight | 1.84 | 1.35 | 2.49 | 0.0001 |
| Obesity | 1.49 | 1.33 | 1.67 | <.0001 |
| Nicotine/tobacco use |  |  |  |  |
| Never | 1.00 |  |  |  |
| Current/former | 1.35 | 1.22 | 1.49 | <.0001 |
| Pre-existing Diabetes |  |  |  |  |
| No | 1.00 |  |  |  |
| Yes | 0.95 | 0.89 | 1.01 | 0.1153 |
| Pre-existing hypertension |  |  |  |  |
| No | 1.00 |  |  |  |
| Yes | 1.34 | 1.21 | 1.48 | <.0001 |
| Pre-existing cardiovascular conditions |  |  |  |  |
| No | 1.00 |  |  |  |
| Yes | 2.62 | 2.46 | 2.79 | <.0001 |
| Previous anthracycline use |  |  |  |  |
| No | 1.00 |  |  |  |
| Yes | 1.30 | 1.05 | 1.60 | 0.0172 |

Table s3. Multivariable Cox Proportional hazard regression for the entire study cohort (n=7330)

(Newly diagnosed cardiovascular adverse events between carfilzomib users and non-users)
